# Characterizing Glycemic Control and Sleep in Adults with Long-Standing Type 1 Diabetes and Hypoglycemia Unawareness Initiating Hybrid Closed Loop Insulin Delivery

**DOI:** 10.1155/2021/6611064

**Published:** 2021-02-12

**Authors:** Susan Kohl Malone, Amy J. Peleckis, Laura Grunin, Gary Yu, Sooyong Jang, James Weimer, Insup Lee, Michael R. Rickels, Namni Goel

**Affiliations:** ^1^Rory Meyers College of Nursing, New York University, New York, NY 10010, USA; ^2^Institute for Diabetes, Obesity, and Metabolism, Perelman School of Medicine, University of Pennsylvania, Philadelphia, PA 19104, USA; ^3^PRECISE Center, Department of Computer and Information Science, School of Engineering and Applied Sciences, University of Pennsylvania, Philadelphia, PA 19104, USA; ^4^Biological Rhythms Research Laboratory, Department of Psychiatry and Behavioral Sciences, Rush University Medical Center, Chicago, IL 60612, USA

## Abstract

Nocturnal hypoglycemia is life threatening for individuals with type 1 diabetes (T1D) due to loss of hypoglycemia symptom recognition (hypoglycemia unawareness) and impaired glucose counter regulation. These individuals also show disturbed sleep, which may result from glycemic dysregulation. Whether use of a hybrid closed loop (HCL) insulin delivery system with integrated continuous glucose monitoring (CGM) designed for improving glycemic control, relates to better sleep across time in this population remains unknown. The purpose of this study was to describe long-term changes in glycemic control and objective sleep after initiating hybrid closed loop (HCL) insulin delivery in adults with type 1 diabetes and hypoglycemia unawareness. To accomplish this, six adults (median age = 58 y) participated in an 18-month ongoing trial assessing HCL effectiveness. Glycemic control and sleep were measured using continuous glucose monitoring and wrist accelerometers every 3 months. Paired sample *t*-tests and Cohen's *d* effect sizes modeled glycemic and sleep changes and the magnitude of these changes from baseline to 9 months. Reduced hypoglycemia (*d* = 0.47‐0.79), reduced basal insulin requirements (*d* = 0.48), and a smaller glucose coefficient of variation (*d* = 0.47) occurred with medium-large effect sizes from baseline to 9 months. Hypoglycemia awareness improved from baseline to 6 months with medium-large effect sizes (Clarke score (*d* = 0.60), lability index (*d* = 0.50), HYPO score (*d* = 1.06)). Shorter sleep onset latency (*d* = 1.53; *p* < 0.01), shorter sleep duration (*d* = 0.79), fewer total activity counts (*d* = 1.32), shorter average awakening length (*d* = 0.46), and delays in sleep onset (*d* = 1.06) and sleep midpoint (*d* = 0.72) occurred with medium-large effect sizes from baseline to 9 months. HCL led to clinically significant reductions in hypoglycemia and improved hypoglycemia awareness. Sleep showed a delayed onset, reduced awakening length and onset latency, and maintenance of high sleep efficiency after initiating HCL. Our findings add to the limited evidence on the relationships between diabetes therapeutic technologies and sleep health. This trial is registered with ClinicalTrials.gov (NCT03215914).

## 1. Introduction

Automated insulin delivery systems and continuous glucose monitoring (CGM) are transforming type 1 diabetes management and improving glycemic outcomes. Insulin pump therapy and CGM are associated with lower hemoglobin A1c (HbA1c) levels compared to insulin injections across all age groups [1]. These clinical benefits have contributed to increase in insulin pump use from 57% to 63% and in CGM use from 7% to 30%, over a six to eight-year period in persons with type 1 diabetes [1]. A 10-fold increase in CGM by children < 12 years old [1] indicates these technologies will become increasingly mainstream for adults in the near future.

Insulin pumps that use hybrid closed loop insulin delivery automatically adjust insulin delivery based on glucose levels. User intervention is required for insulin boluses prior to meals and for correction of hyperglycemia. Hybrid closed loop insulin delivery promises hypoglycemia avoidance because insulin delivery is suspended when glucose levels fall, or are predicted to fall, below a specified threshold. This predictive suspension of insulin delivery feature reduced the frequency of nights with at least one hypoglycemic event from 30% to 18% and the duration of nocturnal hypoglycemic events by 81% compared to nights without the predictive suspension feature activated in a randomized crossover trial [2]. Automated insulin pumps may particularly benefit individuals with type 1 diabetes and hypoglycemia unawareness because they do not experience warning symptoms of low blood glucose. Hypoglycemia unawareness places individuals with type 1 diabetes at greatest risk for severe and life-threatening hypoglycemia [3, 4]. These severe hypoglycemic events are more likely to occur during the night than during the day [5].

One serendipitous benefit of automated insulin pumps may be improved sleep resulting from improved glycemic control and reduced fear of nocturnal hypoglycemia [6, 7]. Following nocturnal hypoglycemia, adults report difficulty returning to sleep, as well as the need to nap and to go to bed early the following day [8]. Few studies have systematically examined the long-term effects of insulin pumps on habitual sleep in adults with type 1 diabetes, and these have used self-report sleep assessments. Six adults reported improved sleep after completing four weeks of closed versus open insulin pump therapy during semistructured interviews [9]. Beato-Vibora et al. found that the percentage of adults reporting poor sleep quality decreased from 49% to 40% three months after initiating hybrid closed loop insulin delivery, but this study did not follow participants beyond three months [10]. On the other hand, those using hybrid closed loop insulin pumps have also reported frequent nocturnal interruptions [11]. Longitudinal studies using objective sleep tracking are needed in persons initiating insulin pump therapy because a significant amount of time is required to acclimate to the use of the new diabetes technology before sleep may be improved and since self-reported sleep changes are often inaccurate [12]. Understanding the relationship between insulin pump use and habitual sleep is particularly important because sleep is increasingly considered a critical factor in diabetes management; indeed, the American Diabetes Association recommends assessing sleep duration and sleep patterns in persons with diabetes [13].

The relationship between sleep and glycemic outcomes in adults with type 1 diabetes is inconsistent. Inadequate sleep, characterized as short, irregular, fragmented, or of poor quality, has been associated with poor glycemic control in adults with type 1 diabetes in some, but not all, studies (14 for review). Shorter versus longer sleep has been associated with higher HbA1c levels and greater glycemic variability in some [14–17] but not all [18–20] studies. More variable sleep durations and sleep midpoints have been associated with higher HbA1c levels and insulin requirements [19, 21, 22]. Fragmented sleep has also been associated with higher HbA1c levels [23]. Longer versus shorter sleep onset latency has been associated with greater glycemic variability [23]. Lastly, poor sleep quality has been associated with higher HbA1c in some adults with type 1 diabetes [16] but not all [23]. Despite this extant evidence, the impact of initiating and acclimating to insulin pump use on objectively measured habitual sleep is absent. As the use of insulin pumps increasingly becomes a standard of care in type 1 diabetes management, a fortuitous opportunity for improving sleep may be possible.

The purpose of this single-arm longitudinal study is to describe accelerometry-estimated sleep and concurrently measured glycemic control at baseline and after initiating a hybrid closed loop insulin delivery system at 3 months, 6 months, and 9 months in adults with long standing type 1 diabetes (>10 years) and hypoglycemia unawareness. The repeated measure design of this study accounts for the likelihood that associations between habitual sleep and glycemic control are individualistic [24]. Moreover, this design also allowed participants to serve as their own control because their baseline data were collected prior to the initiation of a hybrid closed loop insulin delivery system.

## 2. Materials and Methods

### 2.1. Study Participants

Participants were recruited between 2017 and 2020 from various University of Pennsylvania Health System diabetes practices, referrals from local endocrinology providers, or from responses to Penn Institute for Diabetes, Obesity and Metabolism website postings, ClinicalTrials.gov postings, or an IRB-approved secure on-line system (iConnect). All participants provided written informed consent prior to study procedures. Adult participants between 25 and 70 years old were selected based on having long standing, C-peptide negative type 1 diabetes (>10 years) that was diagnosed prior to 40 years of age. Participants were also required to have hypoglycemia unawareness and to be involved in intensive diabetes management, defined by multiple dose insulin injections or continuous subcutaneous insulin infusion with >3 times/day self-blood glucose monitoring and ≥3 clinic evaluations with an endocrinologist or diabetes nurse practitioner during the previous 12 months. Hypoglycemia unawareness was determined by a Clarke score ≥ 4 and either a hypoglycemia severity (HYPO score) ≥ 90th percentile or a composite of a HYPO score ≥ 75th percentile and a glycemic lability (lability index) ≥ 75th percentile. Hypoglycemia exposure was confirmed with >5% of sensor glucose levels < 60 mg/dL and at least one episode of nocturnal hypoglycemia during seven days of blinded CGM. Participants were excluded for insulin requirements ≥ 1.0 units/kg/day, HbA1c ≥ 10%, untreated proliferative diabetic retinopathy, and active cardiovascular, liver, or kidney disease. Additional details are available at ClinicalTrials.gov (NCT03215914).

### 2.2. Study Procedures

Study procedures included a multistage screening phase and an 18-month intervention phase. The screening process began with a history and physical examination that included fasting serum biochemistries, HbA1c, and several hypoglycemia surveys. Retained participants wore a blinded CGM (iPro 2) or their current CGM and a wrist accelerometer (Actigraph GT3X) for seven days. No changes were made to the insulin delivery modality. CGM and accelerometry data were downloaded at the end of the 7-day period to confirm ongoing eligibility. Retained participants wore the CGM (Medtronic MiniMed 670G) without automated features to assess tolerability and compliance and wore the wrist accelerometer for two weeks.

Participants meeting all eligibility criteria and confirming tolerability and compliance with using the CGM and insulin delivery system were trained on using the automated features of the MiniMed 670G system. After one week, participants were transitioned to auto mode. The intervention phase began when the insulin pump was placed in predictive suspension mode. Weekly phone calls were scheduled with the study team to review uploaded insulin dosing, glucose sensor, and glucometer data during the first month. Participants returned for monthly visits through 6 months and then returned at 9 months for review of the CGM and insulin delivery data.

Participants wore an accelerometer for at least two weeks preceding their 3, 6, and 9-month visits. HbA1c levels also were measured at the 3, 6, and 9-month visits. Participants also completed four-week glucose logs and several hypoglycemia surveys at their 6-month visit.

### 2.3. Data Collection and Measures

#### 2.3.1. Glycemic Control

Glycemic control was estimated from HbA1c and CGM data. HbA1c provides a 2 to 3-month average of pre- and postprandial glucose levels [25], and it was calculated from whole blood samples using high performance liquid chromatography (Primus CLC330; Tosoh A1c 2.2 Plus). Interassay coefficients of variation (CV) were <2.54%. CGM uses an electrochemical subcutaneous sensor to estimate interstitial glucose readings every 10 seconds, and glucose estimates were averaged every five minutes. CGM sensor accuracy was assessed at each study visit [26]. CGM data were used to estimate glycemic control during at least a 1-week monitoring period at baseline or run-in and at least a 2-week monitoring period at 3, 6, and 9 months with matching accelerometry data to identify daytime and nighttime periods. CGM data were used to calculate the following metrics: mean sensor glucose, glucose standard deviation (SD), and glucose coefficient of variation (CV), and the percent of time sensor glucose was below range (<54 mg/dL, <60 mg/dL, <70 mg/dL), in range (70 mg/dL-180 mg/dL), and above range (>180 mg/dL, >250 mg/dL) using the HypoCounts software (version 2.0; PRECISE Center, University of Pennsylvania, Philadelphia PA). This software enables integration of accelerometer (see below) and CGM data in order to separate daytime and nighttime defined by sleep onset and sleep offset.

#### 2.3.2. Reduced Hypoglycemia Awareness Was Assessed Using the Clarke Score, the Hypoglycemia Severity (HYPO Score), and Glycemic Lability (Lability Index)

The Clarke score was derived from a reliable and valid 8-item survey used to estimate participants' symptom awareness of hypoglycemia [27]. Participants responded to queries about the frequency of hypoglycemic episodes in the past month and year and their symptomatic responses to hypoglycemia. Responses were scored as “R” for reduced awareness or “A” for aware. Four or more “R” responses indicated reduced awareness [27].

Hypoglycemia severity (HYPO score) estimates the frequency, severity, and degree of hypoglycemia unawareness. The HYPO score was calculated by combining participants' recollection of hypoglycemic episodes and awareness of hypoglycemic symptoms over the previous year with data from four-week blood glucose records. Blood glucose values were used to identify and quantify episodes of serious, clinically significant hypoglycemia (<54 mg/dL). Higher HYPO scores indicate more problematic hypoglycemia [28]; HYPO scores between 423 and 1,046 indicate moderate hypoglycemia problems; scores ≤ 423 indicate no hypoglycemia problems, while scores ≥ 1,047 indicate severe hypoglycemic problems [28]. The reliability and validity of the HYPO score have been established [29].

The lability index estimates changes in glucose over time [28]. Four weeks of glucose records were used to calculate a lability index for each week using the formula described by Ryan et al. [28]. Higher lability index scores indicate less stable glucose levels [28]; a lability index > 433 indicates severe hypoglycemia problems [28]. The lability index has been validated in clinical settings [28].

#### 2.3.3. Sleep

Several dimensions of sleep were estimated from data collected using a well-validated wrist accelerometer (Actigraph wGT3X-BT) [30]. Data collected from wrist-worn accelerometry-estimated rest periods are well established as a method for estimating sleep-wake periods [31]. Wrist movements were recorded at a sample rate of 30 Hz. Data were downloaded using the ActiLife software (version 6.13.3). Data from the actigraphs were collected over at least a 1-week monitoring period at baseline or run-in and at least a 2-week monitoring period at 3, 6, and 9 months. These data were used to calculate various standard sleep variables including sleep duration, sleep onset and midpoint, and sleep efficiency and regularity.

#### 2.3.4. Statistical Analyses

Data from participants completing the 9-month study visit were included in these analyses (*N* = 6); this study is ongoing. Medians and interquartile ranges (IQR) were used to describe the participant's demographics. Means and standard deviations were used to describe participant's accelerometry-estimated sleep characteristics, as well as their BMI, glycemic control, and hypoglycemic measures (Clarke score, HYPO score, and lability index). Paired sample *t*-tests were used to compare means within each individual for changes in glycemic control and sleep characteristics from baseline to 9 months and in hypoglycemic awareness from baseline to 6 months. Because this is an ongoing study, Cohen's *d* effect sizes were used for the primary outcomes, to estimate the magnitude of change from baseline to 9 months or from baseline to 6 months (Clarke score, HYPO score, and lability index), using the following ranges: ≥0.2 small, ≥0.5 medium, and ≥0.8 large [32].

## 3. Results

Participants were mostly White, non-Hispanic, and female (*n* = 5 White, *n* = 1 Asian; *n* = 6 non − Hispanic; *n* = 4 female, *n* = 2 male) with a median age of 58 years (IQR = 19). The median age for type 1 diabetes diagnosis was 15 years old (IQR = 24), and the median duration of type 1 diabetes was 41 years (IQR = 17).

Table 1 presents participants' BMIs, HbA1c levels, and CGM estimates for mean sensor glucose levels and sensor glucose CV; the percentage of time sensor glucose levels were below range (<54 mg/dL, <60 mg/dL, <70 mg/dL), in range (70-180 mg/dL), and above range (>180 mg/dL, >250 mg/dL) and insulin requirements for the monitoring periods. The percentage of time sensor glucose levels were below range, and above range is also reported for participants' accelerometry-determined daytime and nighttime periods. Measures of reduced hypoglycemia awareness for the Clarke scores, HYPO scores, and lability indexes are also presented in Table 1. Medium effect sizes were found for the impact of hybrid closed loop insulin delivery in reducing nocturnal time below range (*d* = 0.64‐0.79), total time below range (*d* = 0.67‐0.70), daytime time below range (*d* = 0.47‐0.52), glucose coefficient of variation (*d* = 0.47), and average daily basal insulin (*d* = 0.48) from baseline to 9 months. Medium to large effect sizes were also found for the impact of hybrid closed loop insulin delivery in reducing the Clarke score (*d* = 0.60), the lability index (*d* = 0.50), and the HYPO score (*d* = 1.06) from baseline to 6 months, see Table 1.

Table 2 presents participants' actigraphy-estimated sleep characteristics over time. Medium to large effect sizes were found for the impact of hybrid closed loop insulin delivery on reducing sleep onset latency (*d* = 1.53), total sleep time (*d* = 0.88), sleep duration (*d* = 0.79), total activity counts (*d* = 1.32), and average awakening length (*d* = 0.46) from baseline to 9 months. Medium to large effect sizes were also found for the impact of hybrid closed loop insulin delivery on delaying sleep onset (*d* = 1.06) and sleep midpoint (*d* = 0.72) from baseline to 9 months. Although not our primary outcome, there was a statistically significant decrease (*t* = 4.48, *p* < 0.01) in accelerometry-estimated sleep onset latency from 4.77 minutes (baseline) to 2.81 minutes (9 months), see Table 2.

## 4. Discussion

The purpose of this longitudinal study was to describe glycemic control and concurrently measured accelerometry-estimated sleep in adults with long standing type 1 diabetes and hypoglycemia unawareness at baseline and after initiating a hybrid closed loop insulin delivery system at 3 months, 6 months, and 9 months. Clinically significant improvements were found for reducing hypoglycemia, glucose variability, and reduced hypoglycemia awareness. These improvements ranged from a medium to large magnitude. There were several changes in sleep after initiating hybrid closed loop insulin delivery. Sleep onset latency, sleep duration, total activity counts, and average awakening length decreased; sleep onset and sleep midpoint were delayed, and high sleep efficiency was maintained after initiating HCL. Collectively, these findings suggest that improvements in glycemic outcomes and changes in sleep accompany hybrid close loop insulin delivery in adults with long standing type 1 diabetes and hypoglycemia unawareness.

Only 21% of adults with type 1 diabetes achieve HbA1c goals [1], and this percentage is lower when diabetes is complicated by reduced hypoglycemia awareness. In this study, the percentage of time that glucose was in target range increased from 66.6% at baseline to 70.0% at 9 months. This increase is comparable to time in range increases reported by others 1 to 6 months after initiating hybrid closed loop insulin delivery [33–35]. Brown et al. reported a time in range increase from 61% to 71% 6 months after initiating hybrid closed loop insulin delivery versus no change for time in range using sensor augmented insulin delivery [36]. These findings are clinically significant because spending more than 70% glucose time in range predicts a HbA1c less than 7%, which is the HbA1c goal for adults with type 1 diabetes [37–39]. Our findings may be particularly important for adults with type 1 diabetes and hypoglycemia unawareness because HbA1c goals are often set higher and time in range goals lower [40] for individuals with a history of severe hypoglycemia [37]. Indeed, time in range increased in the present cohort through a reduction of time spent with hypoglycemia, whereas in previous studies, the increase of time in range was driven by less time spent with hyperglycemia.

Severe hypoglycemia risk is a limiting factor in achieving glycemic goals for individuals with type 1 diabetes and hypoglycemia unawareness. Hypoglycemia severity was reduced as indicated by the decrease in hypoglycemia severity scores after initiating hybrid closed loop therapy. These scores decreased from 909.33 at baseline to 322.67 at 6 months, reflecting a clinically significant reduction in the severity of problematic hypoglycemia [28]. Additionally, sensor glucose CV decreased from 33.5% to 31.3% across 9 months, a finding consistent with other reports of decreases in sensor glucose CV after initiating hybrid closed loop insulin delivery [35, 41]. These findings are clinically important because reducing glucose CV to <33% confers additional hypoglycemia protection compared to the recommended glucose CV of ≤36% [37, 40], which may be particularly critical for adults with hypoglycemia unawareness. The medium effect sizes found for reducing hypoglycemia unawareness and glycemic variability, including Clarke scores and lability indexes, show promise for hybrid closed loop insulin delivery systems in reducing hypoglycemic risk in vulnerable adults with type 1 diabetes and are particularly relevant for adults with type 1 diabetes and hypoglycemia unawareness. These improvements offer the possibility for achieving glycemic goals without increasing life-threatening hypoglycemia risk.

Nocturnal hypoglycemia is associated with a greater likelihood of life-threatening hypoglycemia [42]. In this study, there was a large effect size for the decrease in nocturnal hypoglycemia from baseline to 9 months. This finding is consistent with the reports of others in which there was a decrease in the number of hypoglycemic episodes after initiating hybrid closed loop insulin delivery versus standard insulin pump delivery [43] and a significant decrease in nocturnal hypoglycemia after initiating hybrid closed loop insulin delivery [33].

Increases in insulin requirements are often accompanied by weight gain [44]. Basal insulin decreased after initiating hybrid closed loop insulin delivery in this study and showed a medium effect size. One study reported decreases in the number of correction insulin boluses 3 months after initiating hybrid closed loop insulin delivery compared to sensor augmented pump delivery in a randomized crossover trial [45]. Increases in total daily insulin doses from 47.5 U/d to 50.9 U/d as well as increases in weight from 76.9 kg to 77.6 kg have also been reported after initiating hybrid closed loop insulin delivery [34]. Our finding of a decrease in basal insulin (and no changes in BMI) holds promise in maintaining optimal weight in nonobese adults with type 1 diabetes. Nonetheless, further work is needed to elucidate the relationships between hybrid closed loop insulin delivery with possible changes in insulin requirements.

Our study sample had several dimensions of good sleep at baseline, specifically for duration and timing, as well as excellent sleep efficiency. There were several changes in these sleep dimensions from baseline to 9 months of medium to large magnitude. Sleep duration remained within the 7-8 hours of recommended sleep per night [46], despite a decrease from 8.2 hours at baseline to 7.5 hours at 9 months. Sleep efficiency was excellent throughout the study, ranging from 89.9% to 90.7%. Changes in sleep timing were characterized by a 30-minute delay in sleep onset and sleep midpoint from baseline to 9 months of medium magnitude. Improvements in sleep were characterized by a decrease in sleep onset latency and average awakening length of large and medium magnitude, respectively.

Strengths of this study include the 9-month longitudinal study design and concurrently estimated objective glycemic and sleep outcome measures. This study is limited by the small sample size and limited demographic characteristics of the participants. The current sample size precludes the ability to determine statistical significance in glycemic and sleep changes after initiating hybrid closed loop insulin delivery, and so, we restricted our analysis to the estimation of effect sizes. Moreover, our study was comprised of mostly White females thus limiting the generalizability of our findings to other demographic groups.

## 5. Conclusions

Hybrid closed loop insulin delivery led to clinically significant reductions in hypoglycemia in adults with long standing type 1 diabetes complicated by hypoglycemia unawareness over 9 months. These hypoglycemic improvements were achieved alongside decreases in basal insulin. These findings are particularly relevant because hypoglycemia unawareness presents a unique barrier to achieving glycemic goals in adults with type 1 diabetes. Good sleep was maintained as indicated by several sleep parameters and by excellent sleep efficiency throughout the 9-month study period after initiating HCL. Moreover, some sleep dimensions improved as indicated by shorter sleep onsets and nocturnal awakening lengths after initiating HCL. This finding is important because others have reported greater sleep disturbances after initiating similar diabetes therapeutic devices due to factors such as frequent alarms or device bulkiness. Our findings add to the limited evidence on the relationships between diabetes therapeutic technologies and sleep health. Sleep will remain important to consider as new diabetes therapeutic technologies become increasingly mainstream and approaches to improving sleep quality in adults with long-standing type 1 diabetes are needed.

## Figures and Tables

**Table 1:** BMI and glycemic characteristics at baseline and at 3 months, 6 months, and 9 months after initiating hybrid closed loop insulin delivery and the change values from baseline to 9 months in BMI and glycemic characteristics.

Variable	Baseline Mean (SD)	3 months Mean (SD)	6 months Mean (SD)	9 months Mean (SD)	*t*-test		Effect size^a^
					Baseline to 9 months		
					*t*	*p*	*d*
BMI	24.18 (1.12)	24.54 (1.63)	23.73 (1.41)	24.51 (1.36)	-0.78	0.47	0.26
HbA1c (%)	7.25 (1.33)	7.48 (0.56)	7.60 (1.03)	7.48 (1.06)	-0.64	0.55	0.19
Mean sensor glucose (mg/dL)	147.83 (24.32)	156.33 (23.75)	154.50 (28.56)	153.67 (32.50)	-0.61	0.57	0.20

Percentage of time sensor glucose was in range							
70-180 mg/dL	66.60 (12.80)	68.00 (15.02)	68.40 (21.34)	70.00 (23.48)	-0.43	0.69	0.28

Percentage of time sensor glucose was below and above range (total)							
<54 mg/dL	1.50 (1.93)	0.86 (0.87)	0.37 (0.28)	0.51 (0.68)	1.21	0.28	0.68
<60 mg/dL	2.35 (2.83)	1.38 (1.32)	0.72 (0.33)	0.88 (0.91)	1.32	0.24	0.70
<70 mg/dL	4.86 (5.34)	2.54 (2.00)	2.01 (0.84)	2.22 (1.42)	1.36	0.23	0.67
>180 mg/dL	25.63 (15.20)	27.57 (16.07)	27.88 (20.05)	26.41 (22.33)	-0.13	0.90	0.04
>250 mg/dL	4.66 (4.25)	7.29 (8.01)	6.54 (9.72)	7.58 (12.11)	-0.61	0.57	0.32

Percentage of time sensor glucose was below and above range during the daytime as defined by accelerometry estimated sleep-wake period							
<54 mg/dL	1.25 (1.25)	1.15 (1.02)	0.49 (0.51)	0.67 (0.91)	0.99	0.37	0.52
<60 mg/dL	1.99 (2.05)	1.83 (1.59)	0.91 (0.58)	1.12 (1.22)	1.14	0.31	0.52
<70 mg/dL	4.27 (4.22)	3.37 (2.60)	2.40 (1.20)	2.70 (2.11)	1.21	0.28	0.47
>180 mg/dL	26.50 (17.72)	30.89 (15.15)	32.29 (17.38)	30.84 (22.26)	-1.11	0.32	0.22
>250 mg/dL	4.70 (4.32)	9.37 (9.55)	8.04 (10.37)	8.70 (13.52)	-0.91	0.40	0.40
Percentage of time sensor glucose was below and above range during the nighttime as defined by accelerometry estimated sleep-wake period							

<54 mg/dL	1.99 (3.93)	0.30 (0.72)	0.25 (0.25)	0.21 (0.33)	1.07	0.33	0.64
<60 mg/dL	3.02 (5.16)	0.54 (0.98)	0.47 (0.48)	0.44 (0.51)	1.17	0.30	0.70
<70 mg/dL	5.93 (8.09)	1.00 (1.03)	1.49 (1.14)	1.36 (1.36)	1.27	0.26	0.79
>180 mg/dL	24.26 (15.86)	19.82 (21.98)	20.45 (26.20)	18.35 (24.21)	0.59	0.58	0.29
>250 mg/dL	4.67 (7.24)	6.56 (8.90)	4.17 (8.81)	5.05 (10.06)	-0.07	0.95	0.04

Sensor glucose variability							
Standard deviation	41.86 (20.13)	44.86 (21.07)	42.57 (20.28)	42.00 (19.41)	0.07	0.95	0.01
Coefficient of variation (%)	33.50 (6.37)	33.27 (2.37)	31.83 (1.72)	31.33 (1.51)	0.82	0.45	0.47

Insulin requirements							
Average total daily (U/d)^a^	33.60 (8.47)	34.20 (10.83)	32.20 (9.58)	34.20 (13.74)	-0.19	0.86	0.01
Average daily boluses (U/d)^a^	17.60 (6.47)	18.80 (9.88)	18.20 (7.60)	19.80 (12.28)	-0.82	0.46	0.25
Average daily basal (U/d)^a^	16.00 (5.15)	15.40 (3.51)	14.00 (4.30)	14.40 (4.04)	1.04	0.36	0.48
Clarke score	5.17 (1.17)	n/a	4.50 (1.05)	n/a	1.35	0.24^b^	0.60^b^
HYPO score	909.33 (615.85)	n/a	322.67 (494.35)	n/a	1.69	0.15^b^	1.06^b^
Lability index	351.17 (145.38)	n/a	280.82 (137.18)	n/a	1.24	0.27^b^	0.50^b^

^a^ *n* = 5. ^b^Baseline to 6 months.

**Table 2:** Sleep characteristics at baseline and at 3 months, 6 months, and 9 months after initiating hybrid closed loop insulin delivery and the change values from baseline to 9 months in sleep characteristics.

Accelerometry derived variable	Baseline Mean (SD)	3 months Mean (SD)	6 months Mean (SD)	9 months Mean (SD)	*t*-test		Effect size^a^
					Baseline to 9 months		
					*t*	*p*	*d*
Sleep onset latency (minutes)	4.77 (1.41)	4.05 (0.60)	3.85 (1.99)	2.81 (1.14)	4.48	<0.01	1.53
Sleep onset (hr : min)	22 : 00 (00 : 43)	22 : 22 (00 : 23)	22 : 36 (1 : 15)	22 : 49 (00 : 53)	-1.76	0.14	1.06
Sleep offset (hr : min)	7 : 02 (00 : 38)	6 : 58 (00 : 43)	7 : 18 (00 : 47)	7 : 06 (00 : 46)	-0.24	0.81	0.10
Total sleep time (minutes)	541.54 (33.16)	515.23 (44.47)	518.03 (71.39)	499.02 (59.77)	1.14	0.30	0.88
Sleep duration (minutes)	490.37 (26.03)	465.64 (51.16)	466.47 (68.60)	451.93 (64.05)	1.06	0.34	0.79
Wake after sleep onset (minutes)	46.39 (7.45)	45.54 (12.98)	47.72 (4.97)	44.27 (12.78)	0.62	0.56	0.20
Nighttime awakenings (number)	15.67 (4.46)	16.61 (4.20)	17.57 (4.16)	15.73 (3.91)	-0.04	0.97	0.01
Average awakening length (minutes)	3.31 (0.81)	2.84 (0.69)	2.89 (0.95)	2.97 (0.69)	1.10	0.32	0.46
Sleep fragmentation index	24.85 (6.97)	26.65 (9.47)	25.31 (6.56)	24.79 (9.93)	0.04	0.97	0.01
Total activity counts (number)	32,175.92 (4185.06)	29,503.42 (6974.19)	29,291.94 (6322.80)	25,803.08 (5372.96)	2.17	0.08	1.32
Sleep efficiency (percent)	90.70 (1.14)	90.30 (3.02)	89.86 (1.53)	90.35 (3.46)	0.34	0.75	0.14
Sleep midpoint (hr : min)	2 : 32 (00 : 38)	2 : 42 (00 : 27)	3 : 00 (00 : 51)	3 : 00 (00 : 37)	-1.90	0.12	0.72

^a^Baseline to 9 months.

## Data Availability

The original/source data for the analyses and tables reported in the paper will be made available from the corresponding author upon request at the time of publication.
